# Facial Nerve Injury after Extracapsular Dissection for Benign Parotid Tumors with and without Intraoperative Monitoring: A Retrospective Study of a Single Center

**DOI:** 10.3390/diagnostics14182017

**Published:** 2024-09-12

**Authors:** Maria Giulia Cristofaro, Walter Colangeli, Francesco Ferragina, Giuseppe Tarallo, Angelo Ruggero Sottile, Maria Grazia Ioppolo, Antonella Arrotta, Ida Barca

**Affiliations:** 1 Maxillofacial Surgery Unit, Department of Experimental and Clinical Medicine, Renato Dulbecco Hospital, Magna Graecia University of Catanzaro, Viale Europa, 88100 Catanzaro, Italy; colangeli@tin.it (W.C.); francesco.ferragina92@gmail.com (F.F.); giuseppe.tarallo92@gmail.com (G.T.); angelo.sottile.1991@gmail.com (A.R.S.); magioppolo@gmail.com (M.G.I.); barca.ida@gmail.com (I.B.); 2 Department of Medical and Surgical Sciences, Anesthesia and Intensive Care, Renato Dulbecco Hospital, Magna Graecia University of Catanzaro, Viale Europa, 88100 Catanzaro, Italy; antonella.arrotta@hotmail.it

**Keywords:** benign parotid tumor, extracapsular dissection, postoperative facial nerve paralysis, intraoperative facial nerve monitoring

## Abstract

Background: Facial nerve injury (FNI) is the most common complication of parotid surgery and manifests as FN paralysis. The use of intraoperative facial nerve monitoring (IFNM) is becoming an established intraoperative aid for surgeons, assisting in the identification of the location and dissection of the facial nerve trunk or branches. The postoperative outcomes of parotid surgery with and without monitoring have been addressed in only a limited number of studies. Objective: The objective of this study is to evaluate the incidence of postoperative paralysis in patients undergoing extracapsular dissection (ED) for benign parotid tumors concerning the use or non-use of IFNM. Materials and Methods: The retrospective study was conducted at the Maxillo-Facial Department of the Magna Graecia University of Catanzaro. The patients were divided into two groups: Group 1 consisted of patients who underwent surgery without IFNM (1 January 2015 to 31 December 2018); Group 2, on the other hand, consisted of patients who underwent surgery with IFNM (1 January 2019 to 31 December 2022). Group 2 employed the Nerve Integrity Monitor (Medtronic’s NIM^®^). To classify the FN function, we employed the modified House–Brackmann classification system. To evaluate the dependence between the “use of IFNM” and “postoperative paralysis”, a descriptive analysis was conducted, including applying the Chi-squared test and calculating the Pearson correlation. Subsequently, a binary logistic regression model was applied to further evaluate the correlation between the latter. The level of statistical significance was set at *p* < 0.05. Results: A total of 276 patients were included in the study: 120 subjects were assigned to Group 1 (43.5%, comprising 60 men and 60 women) and 156 subjects were assigned to Group 2 (56.5%, comprising 93 men and 63 women). In 91.7% of the cases (n. 253, precisely 105 in Group 1 and 148 in Group 2), no FNI occurred. In 8.33% of the cases (n. 23, specifically 15 in Group 1 and 8 in Group 2), postoperative paralysis was observed. Of these subjects, only two in Group 1 had permanent paralysis (8.69%); therefore, 91.31% had transient paralysis. As a result, 91.31% of the subjects exhibited transient paralysis. In the case of FNI, 78% of the cases involved the marginal mandibular branch (n. 18), 13% involved the temporo-zygomatic branch (n. 3), and 7% involved more than one branch (*n* = 2). The results of the multivariable binary logistic regression analysis demonstrated that the use of IFNM was a statistically significant influencing factor, with an estimated reduction in postoperative paralysis of approximately 62% (OR 0.378; 95% CI: 0.155–0.92). In Group 2, the occurrence of transient complications was significantly reduced (OR 0.387; 95% CI: 0.149–1.002 with *p* < 0.05). Discussion and Conclusions: The use of IFNM in the ED for benign parotid tumors significantly reduces the rate of FNI and, consequently, postoperative FN paralysis. On the other hand, the use of monitoring systems must not replace the experience and anatomical knowledge of the surgeon.

## 1. Introduction

Parotid tumors represent 1% to 3% of all primary head and neck cancers, with benign histopathological characteristics present in 70% to 90% of cases [1,2,3,4,5,6]. Most of these benign lesions are pleomorphic adenomas (PAs), followed by Warthin tumors (WTs) and, less frequently, oncocytomas, basal cell adenomas, and others [4]. Approximately 60% of cases are attributed to PAs. Surgical treatment of these tumors has changed a lot in the last century. Before 1930, parotid surgery aimed to limit facial nerve (FN) damage. Intra-capsular enucleation (ICE) was the most common procedure [7]. This technique has a 20–40% recurrence rate [8,9]. Superficial parotidectomy (SP) has been demonstrated to reduce recurrence to approximately 2% [10]. However, SP is associated with an elevated risk of complications, including FN paralysis, Frey syndrome, salivary fistula, and aesthetic concerns. Total parotidectomy (TP) has been shown to further reduce the rate of relapses, yet it is also associated with an increased incidence of postoperative complications.

Postoperative complications can be classified into two main categories: locoregional and systemic. Locoregional complications include edema of the surgical site, wound infections, and bleeding, whereas systemic complications include facial nerve injury (FNI), FN paralysis, dysesthesia of the great auricular nerve, salivary fistula, Frey syndrome, capsular rupture, and relapse.

FNI represents the most prevalent complication of parotid surgery, manifesting as paralysis. FNI is a consequence of several factors, including nerve transection, clamping, electrothermal injury, stretching or entrapment, stapling, aspiration trauma, and/or ischemia. In the existing literature, transient FN paralysis is observed in 15–60% of parotid gland surgeries, with a higher incidence reported for TP compared to SP. The occurrence of permanent paralysis is less prevalent, with an estimated range of 2.5 to 5% [1,2]. Re-intervention for recurrence is associated with an increased risk of paralysis. This phenomenon is attributed to tissue fibrosis and is observed in approximately 100% of cases in the immediate postoperative period. FNI can lead to significant functional impairment and medical/legal disputes. The surgical treatment of the parotid gland has undergone a significant evolution; the first description of the FN was provided by Velpeau in 1830. The initial studies examining the monitoring of the FN during parotid surgery were published in the early 1990s. The objective of contemporary surgical techniques is to achieve a minimal incision with optimal visualization of the surgical field, tumor excision with secure margins, and a low incidence of postoperative complications with a particular emphasis on preserving the function of the facial nerve. To achieve these goals, electrophysiological monitoring of the facial nerve is becoming an established intraoperative tool to assist the surgeon in localizing and dissecting the trunk or its dividing branches. The existing literature on intraoperative facial nerve monitoring (IFNM) is limited and often based on small samples. The FNI rate is contingent upon a multitude of factors, including the experience of the surgical team, the dimensions of the tumor, its anatomical location, and its histological differentiation [3,4,5]. In extracapsular dissection (ED), it is not necessary to pre-identify the root of the facial nerve. Intraoperative monitoring is essential for the identification and preservation of terminal branches [6,7,8,9]. In contrast, in cases of SP or TP, it is imperative to identify the FN trunk and stimulate it after surgery. This is particularly crucial for medical/legal purposes. In 2017, Quer et al. considered a range of parameters, including size, site, and histopathology, to determine the extent of surgery in the treatment of benign parotid tumors. They proposed establishing a four-category classification system by selecting a cut-off diameter of 3 cm [10]. Accordingly, their classification is based on size and site, in accordance with the proposal put forth by the European Salivary Gland Society (ESGS) [11]. The authors put forth the following categorization system: Category I is reserved for tumors measuring ≤ 3 cm, exhibiting superficial (outer surface) characteristics, and situated close to the parotid borders. Category II is designated for tumors ≤ 3 cm, displaying deep characteristics or a distance from the parotid borders. Category III is assigned to tumors > 3 cm that involve two levels, in accordance with the ESGS proposal [8]. Category IV is allocated to tumors > 3 cm that involve more than two levels. For example, they reserve the designation of ED for pleomorphic adenomas measuring 3 cm or less that are mobile and situated close to the parotid borders (category I) and for categories I and II of Warthin’s tumors.

In this retrospective study, the authors evaluated the incidence of postoperative paralysis in patients undergoing ED treatment for benign parotid tumors from 2016 to 2023, with particular attention to the role of IFNM in this context.

## 2. Materials and Methods

The retrospective study was conducted at the Maxillo-Facial Unit of the Magna Graecia University of Catanzaro. The data analyzed pertain to patients who underwent parotid surgery before the introduction of IFNM (1 January 2015–31 December 2018). The data were compared with those of patients undergoing surgery in the period in which the use of IFNM was standardized, during which the surgeon had acquired sufficient experience with the device to be aware of possible setting errors (1 January 2019 to 31 December 2022). This study was approved by the Ethics Committee of the University Magna Graecia of Catanzaro (protocol number 146/2016) and informed consent was obtained from patients.

To mitigate the potential for selection bias, only cases that underwent surgical treatment by two experienced senior surgeons (MGC and IB) were included in the analysis.

A database of all patient information was constructed using Microsoft Excel software 2017 version (Redmond, WA, USA). The patients were divided into two groups:

- Group 1: the surgical procedures were performed without the use of IFNM.
- Group 2: the surgical procedures were performed with the use of IFNM.

The data set comprised demographic information, details of surgical procedures performed, comorbidities, histological diagnoses, and postoperative FN function. Subsequently, data about the recovery or the permanence of the facial paralysis were gathered through clinical and/or telephonic follow-up. In this context, the term “permanent paralysis” is used to describe any degree of facial weakness that persists for a minimum of six months following the surgical procedure.

Patients were evaluated according to the following inclusion criteria:

- Patients of both sexes over the age of 18;
- Patients with postoperative histological diagnosis of benign parotid neoplasms with a mean lesion size of 3.0 ± 0.5 cm;
- Patients undergoing parotid surgery with ED technique.

The following exclusion criteria were applied:

- Patients with postoperative histological diagnosis of malignant parotid neoplasms;
- Patients who have requested a surgical procedure other than ED;
- Patients with non-neoplastic parotid pathologies (retention cysts, sialadenitis, sialolithiasis);
- Patients with recurrent neoplasms;
- Patients with preoperative FN deficit.

The surgical procedure was timed from the moment of incision to the conclusion of suturing, as documented in the anesthetic record. The time required for the installation of the monitoring equipment was neither recorded nor considered in the scheduling of the surgical procedure. Nevertheless, the configuration of electrodes and the subsequent testing typically necessitate a preparation period of less than five minutes. The analysis of the FN function was based on a daily evaluation conducted by the resident or surgeon responsible for noting the FN function in the patient’s medical record. This evaluation was carried out preoperatively, postoperatively on the first day, and remotely, with photographic documentation also provided.

To categorize the FN function, we employed the modified House–Brackmann classification system. To reduce the potential for observer bias in patient records, we elected to group Grade III with Grade IV and Grade V with Grade VI of the House–Brackmann classification. This resulted in a scoring system of levels I to IV of dysfunction, where I indicated no dysfunction, II indicated mild dysfunction, III indicated moderate dysfunction, and IV indicated severe dysfunction (Table 1) [12].

The House–Brackmann classification was employed at three distinct time points: immediately postoperatively, at 3 months, and at 6 months. This was done to distinguish between persistent and transient conditions.

### 2.1. Surgical Technique

In both patient groups, the ED was performed under general anesthesia in line with the previously established standard practice procedures in our clinical setting. One hour before surgery, patients were administered midazolam intravenously at a dosage of 1 to 5 mg. Following the administration of adequate preoxygenation and denitrogenization, anesthesia was induced with propofol 2 mg/kg intravenously (i.v.) as a bolus (for sedation) in association with a single reduced dose of rocuronium (0.3 mg/kg). Analgesia was ensured through the continuous infusion of remifentanil at a rate of up to 1 mcg × kg/min. The ED was conducted in line with the established methodology. The incision was made in consideration of the natural folds of the face and neck flexion (Redon incision), beginning in the preauricular region and extending up to the ear lobe insertion, continuing along the anterior margin of the mastoid, and then posteriorly along the mandibular angle. The integrity of the tumor capsule was maintained with great care by performing a wide excision of the parenchyma surrounding the wound (approximately 2–3 mm from the tumor), without identification of the common trunk of the facial nerve. This procedure is indicated for benign tumors with a maximum diameter of 3.0 ± 0.5 cm. In Group 2, the device was the Nerve Integrity Monitor (NIM^®^) (Medtronic, Fridley, MN, USA), which is one of the most widely utilized monitoring devices in thyroid surgery. The device provides audio-visual information based on electromyographic (EMG) activity resulting from intraoperative nerve stimulation. The device is composed of two main components: a recording electrode and a monopolar or bipolar nerve stimulation probe, which is connected to a pulse generator. The recording electrode is used to investigate a variable tissue volume, while the grounding electrode, which is typically positioned on the patient’s shoulder close to the monitor unit or on the patient’s sternum, serves to reduce electrical interference. Four recording electrodes are utilized: one for the frontal muscle, which is inserted in the lower frontal region; one for the Orbicularis oculi muscle (temporal and zygomatic nerve), which is inserted in the infraorbital area; one for the Orbicularis oris muscle (buccal nerve), which is inserted at the upper lip; and one for the mental muscle, which is inserted at the lower lip. These electrodes are subcutaneously inserted into the muscles after the area is cleaned with alcohol-soaked swabs.

All electrode ends are connected, following the specified color code, to the receiving console, which transmits the electromyographic response to the monitor.

The pulse generator can be employed as a navigational instrument to estimate the distance to the nerve. Stimulation of the facial nerve at an average intensity of 1 mA results in a displacement of 2.20 ± 0.76 mm. This enables the distance from the main branches of the facial nerve to be predicted. The mechanical manipulations evoke high-frequency electromyographic potentials which are associated with audio signals. These signals alert the surgeons. The nerve stimulation probe, set to a range of 1.5–2 mA, was employed to perform nerve mapping. Once the FN trunk has been successfully identified, the stimulation energy is then reduced to a range of 0.5–0.8 mA [13]. Upon completion of the intervention, the system generated a report file in PDF format, which provided the option to view the electromyographic traces.

Following the surgical procedure, patients were observed for potential locoregional postoperative complications (such as edema, wound infection, or hemorrhage) and systemic postoperative complications (such as dysesthesia of the great auricular nerve, salivary fistula, and Frey Syndrome).

### 2.2. Statistical Analysis

The Excel database was subjected to statistical analysis using IBM SPSS software version 19. The data regarding the postoperative outcomes of patients who underwent surgery without monitoring were compared with those of patients who underwent surgery under monitoring.

We used descriptive analyses, specifically the Chi-squared test and Pearson correlation index, to see if there is a link between the use of NIM and postoperative paralysis. Subsequently, we used the binary logistic regression model to further assess the correlation between the variables. The level of statistical significance was set at *p* < 0.05.

## 3. Results

A total of 338 patients underwent ED for benign tumors of the parotid gland throughout the two analyzed periods. Of the total number of patients, 276 (81.7%) met the inclusion criteria. Of these, 153 (55.4%) were male and 123 (44.6%) were female.

Of the 276 patients, 120 (43.5%) were assigned to Group 1, of whom 60 (50%) were male and 60 (50%) were female. The remaining 156 patients (56.5%) were assigned to Group 2, of whom 93 (59.6%) were male and 63 (40.4%) were female. These data are presented in Table 2.

The mean age was 56.86 ± 15.5 for Group 1 and 54.23 ± 14.76 for Group 2 (Table 3).

The descriptive analysis of the postoperative paralysis demonstrated that 253 of the 276 patients (91.7%) did not report experiencing postoperative paralysis. In particular, 105 (87.5%) subjects were observed in Group 1, while 148 (95%) were observed in Group 2. Twenty-three subjects exhibited postoperative paralysis. Of these, 15 cases (12.5%) were observed in Group 1 and 8 cases (5%) in Group 2. Two subjects from Group 1 (0.7%) exhibited permanent paralysis, while twenty-one subjects (7.6%, thirteen from Group 1 and eight from Group 2) exhibited transient paralysis. These data are presented in Table 4.

On the day following surgery, 15 subjects in Group 1 exhibited varying degrees of paralysis. Six subjects demonstrated grade I dysfunction, four exhibited grade II dysfunction, three exhibited grade III dysfunction, and two exhibited grade V dysfunction. Of the eight subjects in Group 2, five exhibited grade I dysfunction, while three demonstrated grade II dysfunction.

The data after the three- and six-month periods are presented below. The two subjects with grade V dysfunction (all from Group 1) exhibited persistent paralysis. The three subjects with grade III dysfunction (all from Group 1) demonstrated paralysis during the resolution phase. The seven subjects with grade II dysfunction (four from Group 1 and three from Group 2) no longer exhibited any dysfunctions. The eleven subjects with grade I dysfunction (six from Group 1 and five from Group 2) no longer exhibited any dysfunctions. These data are presented in Table 5.

The relationship between the use of the NIM surgical monitoring device and the occurrence of paralysis was confirmed through the application of the Chi-squared test, which revealed a statistically significant correlation (*p*-value < 0.05). This was particularly evident in cases of transient paralysis. Subsequently, the Pearson correlation coefficient ρ was calculated, which yielded a negative value, indicating an inverse correlation between the use of the NIM and the presence of paralysis (Table 6).

The incidence of postoperative FN paralysis was 8.3% (n. 23), that of transient paralysis was 7.6% (n. 22), and that of permanent paralysis was 1.1% (n. 2). The incidence of postoperative paralysis with the use of IFNM decreased from 12.5% to 5%, representing a reduction of 7.5%. Consequently, one case of paralysis was avoided for every thirteen patients who underwent surgery with the IFNM.

In the subjects who exhibited paralysis, 78% involved the mandibular marginal branch (n. 18), 13% involved the temporo-zygomatic branch (n. 3), and 7% involved more than one branch (n. 2). The Chi-squared test did not reveal any statistically significant differences in the distribution of branches of the FN affected by paralysis based on the use of NIM (Table 7).

A descriptive analysis was conducted to examine the relationship between the different histotypes of tumors operated on in the two reference periods and the incidence of postoperative paralysis.

The most prevalent histological type was pleomorphic adenoma (n. 130, 48%), followed by Warthin’s tumor (n. 122, 44%). Most cases of postoperative paralysis involved Warthin tumors, with 11 cases identified.

No notable discrepancy in the prevalence of postoperative paralysis was observed across the different histological categories of neoplasia (Table 8).

Concerning locoregional postoperative complications, edema was observed in 234 subjects (84.78%) and resolved on average after five days. Conversely, conspicuous bleeding was noted in 8 subjects (2.9%) and wound infection was noted in 63 subjects (22.82%). Infections were treated with antibiotics, while edema was managed with corticosteroids only when deemed clinically significant. The minor hemorrhages were not treated; however, the more significant ones were treated by the placement of metal clips or electrodiathemocoagulation.

Concerning the systemic postoperative complications, dysesthesia of the great auricular nerve was observed in 182 subjects (65.94%), salivary fistula in 31 subjects (11.23%), and Frey syndrome in 5 subjects (1.8%). Dysesthesia of the great auricular nerve was subjected both to qualitative and quantitative analysis. A qualitative analysis was conducted using tests to assess tactile and thermal sensitivity, the ability to locate the stimulus, and the capacity to distinguish between a sharp and a blunt tip. A quantitative analysis was also conducted to document the ability to discriminate between two points, both statically and dynamically. This was observed over time and potentially treated with drugs that target neutrophils and/or cortisol. All cases of salivary fistulas were treated with a conservative approach, whereas Frey syndrome was treated with a subcutaneous injection of botulinum toxin.

A multivariable binary logistic regression analysis was conducted to verify which factors had influenced the incidence of postoperative paralysis. The analysis revealed that the use of IFNM was a statistically significant factor influencing the incidence of postoperative paralysis, reducing it by approximately 62% (OR 0.378; 95% CI: 0.155–0.925 with *p* < 0.05), particularly in the case of transient paralysis (OR 0.387; 95% CI: 0.149–1.002 with *p* < 0.05). Conversely, no statistically significant reduction in the incidence of permanent postoperative paralysis was observed. However, the number of cases was insufficient to guarantee a reliable analysis. Similarly, the regression model demonstrated that sex, the average age of the patients, and the types of neoplasia considered were not statistically significant factors influencing the number of cases of postoperative paralysis (Table 9).

## 4. Discussion

The objective of surgical intervention for benign parotid gland tumors is to achieve the optimal result in terms of tumor removal, risk of recurrence, and postoperative outcome while maintaining the function of the FN. Surgical procedures for parotid gland tumors exhibit variability due to the tumor’s location, size, histological type, and the course of the FN branches. A large superficial tumor in the inferior portion of the superficial lobe presents fewer surgical challenges than a small malignant tumor near the FN trunk. In the existing literature, transient paralysis of the FN is identified as a potential complication following parotid surgery, with an incidence ranging from 15 to 66%. The reported rate of transient paresis following superficial parotidectomy is 15 to 25%, while the incidence rises to 20 to 50% following total parotidectomy. The incidence of permanent FN paralysis is reported to be between 5% and 10%, with some case series indicating a higher prevalence of up to 100% in cases of recurrent neoplasms [1,2,14].

For this reason, it is exceedingly challenging to develop objective studies that can accurately and consistently evaluate the efficacy of intraoperative facial nerve monitoring during parotid surgery.

In cases of tumors of a size ≤ 3 cm, ED may be the optimal surgical technique to reduce postoperative complications. This approach nevertheless guarantees meticulous dissection, with a margin of at least 1–2 mm of normal glandular tissue, obviating the necessity for pre-identification of the main trunk of the FN.

The utilization of intraoperative monitoring serves to assist the surgeon in identifying the FN, to alert in the event of inadvertent manipulations or stimulations during the dissection, to delineate the trajectory of the nerve, and to project the potential outcome of the nerve function after the procedure. It is hypothesized that the protective effect for transient paralysis is due to the imminent warning of stretch, compression, or thermal damage, which reduces events that can cause edema and alter local microcirculation, with consequences for nervous health. Conversely, an unmonitored dissection can lead to more serious damage to small hidden nerve branches [15].

Nevertheless, the numerous factors that contribute to false positives and false negatives render the intraoperative application of this method a matter of contention and ongoing debate. The use of monitoring may engender a false sense of security in the surgeon, who may perform a nerve dissection with diminished accuracy [16,17]. It is therefore imperative that the team responsible for utilizing the monitoring be adequately trained, as excessive use of the stimulation probe could result in iatrogenic stupor due to excessive discharges.

A recent meta-analysis, which included prospective and retrospective studies, reports a 42.7% reduction in postoperative transient facial nerve deficits and a 7.8% reduction in the incidence of permanent deficits. However, some bias from retrospective cohorts could not be ruled out [18].

A further meta-analysis (Sood et al., 2015) indicates a significant 47% reduction in transient paralysis, with no discernible impact on the patient’s outcome [19].

In the various case studies reported in the literature, there is a lack of consistency in how cases are distinguished based on key variables. These variables include the histological type of the tumor (malignant vs. benign), size (less than or greater than 3 cm), morphology, location, the inflammatory state of the parotid gland, previous operations on the same parotid, the type of surgery performed, clinical status of the patient, sex, and age of the patient, and experience of the surgeon. This lack of consistency makes the results susceptible to multiple biases [20]. It is important to note that malignant tumors have the potential to infiltrate branches of the FN, necessitating a radical treatment strategy. It is therefore not appropriate to compare malignant tumors with benign tumors or to use them as a means of evaluating the effectiveness of intraoperative facial nerve monitoring. Consequently, patients with malignant tumors were excluded from this study.

Some authors posit that intraoperative monitoring is advantageous in challenging FN dissections, particularly in patients with large tumors and in revision surgery, where direct visualization may be inadequate. Other studies have demonstrated that surgical times are reduced in parotidectomies performed under monitoring and that the severity of lesions is diminished in the monitored group [21,22,23].

This study presents the most relevant evidence in the scientific literature, demonstrating a significant reduction in the incidence of postoperative paralysis of the facial muscles in patients undergoing parotid surgery with the use of IFNM (−62%, *p*-value 0.033), particularly for transient paralysis. However, the same was not demonstrated for permanent paralysis, due to the limited statistical sample, which represented a limitation in obtaining a reliable result. The authors performed ED, which has a lower incidence of postoperative complications such as FN paralysis [24,25,26]. In our retrospective study, a normal result (House–Brackmann grade I) was obtained by a significantly greater number of patients in Group 2 than in Group 1 three months postoperatively (153% vs. 111%, *p* < 0.001). The multivariable binary logistic regression analysis revealed that the use of IFNM was a statistically significant influencing factor, reducing postoperative paralysis by approximately 62% (OR 0.378; 95% CI: 0.155–0.925 with *p* < 0.05). Specifically, transient cases demonstrated a statistically significant reduction in postoperative paralysis (OR 0.387; 95% CI: 0.149–1.002 with *p* < 0.05). Conversely, no statistically significant reduction in the incidence of permanent postoperative paralysis was observed. However, the number of cases was insufficient to guarantee a reliable analysis. Similarly, the regression model demonstrated that sex, the average age of the patients, and the types of tumors considered were not statistically significant factors influencing the incidence of postoperative paralysis.

Moreover, no notable differences in postoperative paralysis rates were observed based on the patient’s age, sex, or the histological type of benign neoplasm. Our experience indicates that intraoperative monitoring is an effective method for reducing the incidence of transient postoperative paralysis in the emergency department. Further studies are necessary to evaluate the possible effectiveness of IFNM in the treatment of benign tumors of the superficial lobe >3 cm treated with PS, or benign tumors with different locations, which would therefore be treated with different surgical approaches. Moreover, the design of a prospective study will enable the identification of the potential benefits of IFNM, as previously described in the literature, in reducing the severity of lesions and shortening the recovery period from transient postoperative paralysis.

## 5. Conclusions

FN paralysis represents one of the most serious complications that can occur in parotid gland surgery. This retrospective study demonstrated that IFNM represents an effective method for reducing the risk of immediate and late postoperative facial nerve paralysis. The authors strongly recommend the use of IFNM in parotid surgery, particularly for benign lesions treated with ED which above all requires the identification and preservation of the terminal branches of the FN. It is important to note that the use of monitoring systems must not replace the experience and anatomical knowledge of the surgeon.

## Figures and Tables

**Table 1:** The staging of FN function according to the House–Brackmann classification.

Grading	FN Function
I: Normal	No deficit.
II: Mild dysfunction	Slight facial weakness or other mild dysfunction, normal tone and symmetry at rest; complete closure of the eye without effort; slight asymmetry of the mouth when facial movements occur.
III: Moderate dysfunction	No facial weakness with synkinesis and complete eye closure and good forehead movement with effort.
IV: Moderate–severe dysfunction	Obvious facial weakness. Incomplete eye closure, no forehead movement, asymmetrical mouth movement, and synkinesis.
V: Severe dysfunction	Little to no ability to smile, frown, or make other facial expressions. The closure of the eye is incomplete, and there is no forehead movement.
VI: Complete paralysis	No facial motion.

**Table 2:** Frequencies of patients operated in the two groups.

	Operated Patients		
	Group 1	Group 2	Total
Male	60 (50%)	93(59.6%)	153 (55.4%)
Female	60 (50%)	63 (40.4%)	123 (44.6%)
Total	120 (43.5%)	156 (56.5%)	276

**Table 3:** The average age of patients in the two groups.

	Group 1	Group 2	Total	*p*-Value
Patients	120	156	276	
Average age (±DS)	56.86 ± 15.5	54.23 ± 14.76	54.07 ± 15.06	0.1522

The mean lesion size was 3.0 ± 0.5 cm in both groups.

**Table 4:** Postoperative paralysis of FN rates in the two groups.

Postoperative Paralysis Rates	Group 1	Group 2	Total	*p*-Value
Patients without paralysis.	105 (87.5%)	148 (95%)	253 (91.7%)	0.028
Patients with paralysis.	15 (12.5%)	8 (5%)	23 (8.3%)	
Permanent paralysis.	2 (0.7%)	0	2 (0.7%)	0.27
Transient paralysis.	13	8	21 (7.6%)	

**Table 5:** FNI assessed by House–Brackmann classification.

DAY	Group 1	No Facial Paralysis	Group 2	No Facial Paralysis
Day 1	Facial palsy (Gr V): 2/15 Gr I: 6/15 Gr II: 4/15 Gr III: 3/15	105	Gr I: 5 Gr II: 3	148
Days 90	Facial palsy (Gr V): 2/15	111	Gr II: 3	153
Days 180	Facial palsy(Gr V): 2/15 Gr III: 3/15	115		156

**Table 6:** Postoperative paralysis rates in the two groups.

Postoperative Paralysis Rates	Group 1	Group 2	Total (%)	*p*-Value	Index ρ
Patients without paralysis	105 (87.5%)	148 (95%)	253 (91.7%)	0.028	−0.132
Patients with paralysis	15 (12.5%)	8 (5%)	23 (8.3%)	0.044	−0.121

**Table 7:** The incidence of FN damage in the two study groups.

Facial Nerve Branches				
	Group 1 (%)	Group 2 (%)	Total (%)	*p*-Value
Temporal-zygomatic nerve	2 (13%)	1 (13%)	3 (13.04%)	0.95
Marginalis Mandibulae nerve	11 (74%)	7 (87%)	18 (78.26%)	0.43
Deficiency of more than one branch	2 (13%)	0	2 (8.7%)	0.21

**Table 8:** Incidence of postoperative paralysis relating to histological type.

Histological Types	Group 1	Group 2	Total (%)	*p*-Value
*Pleomorphic adenoma*	61	69	130 (48%)	
Postoperative paralysis	3	3	8	0.88
*Warthin tumor*	49	73	122 (44%)	
Postoperative paralysis	7	4	11	0.10
*Oncocytomas*	3	5	8 (3%)	
Postoperative paralysis	1	1	2	0.67
*Myoepithelioma*	1	1	2 (0.7%)	
Postoperative paralysis	1	0	1	0.16
*Lipoma*	6	8	14 (5%)	
Postoperative paralysis	1	0	1	0.23

**Table 9:** Regression model.

	OR	Lower Limit	Upper Limit	*p*-Value
Use of IFNM G2	0.378	0.140	0.885	0.027
Sex (F)	0.565	0.207	1.542	0.265
Average age	1.033	0.999	1.069	0.060
Histological type				0.310

## Data Availability

The data are available in the hospital archive of the Maxillo-Facial Unit of Catanzaro.
